# Validating Left Ventricular Filling Pressure Measurements in Patients with Congestive Heart Failure: CardioMEMS™ Pulmonary Arterial Diastolic Pressure versus Left Atrial Pressure Measurement by Transthoracic Echocardiography

**DOI:** 10.1155/2018/8568356

**Published:** 2018-07-15

**Authors:** Sunit Tolia, Zubair Khan, Gunjan Gholkar, Marcel Zughaib

**Affiliations:** Department of Cardiology, Providence-Providence Park Hospital, Michigan State University College of Human Medicine, 16001 W. Nine Mile Road, Southfield, MI 48075, USA

## Abstract

**Background:**

Routine ambulatory echocardiographic estimates of left ventricular (LV) filling pressures are not cost-effective and are occasionally fraught with anatomic, physiologic as well as logistical limitations. The use of implantable hemodynamic devices such as CardioMEMS Heart Failure (HF) System has been shown to reduce HF-related readmission rates by remote monitoring of LV filling pressures. Little is known about the correlation between CardioMEMS and echocardiography-derived estimates of central hemodynamics.

**Methods:**

We performed a prospective, single-center study enrolling seventeen participants with New York Heart Association functional class II-III HF and preimplanted CardioMEMS sensor. Simultaneous CardioMEMS readings and a limited echocardiogram were performed at individual clinic visits. Estimated left atrial pressure (LAP) by echocardiogram was calculated by the Nagueh formula. Linear regression was used as a measure of agreement. Variability between methods was evaluated by Bland–Altman analysis.

**Results:**

Mean age was 74 ± 9 years; 59% (10/17) were males. LV systolic dysfunction was present in 76% (13/17) of subjects. Mean PAdP was 18 ± 4 mmHg and 19 ± 5 mmHg for CardioMEMS and echocardiographic-derived estimates, respectively, with a significant correlation between both methods (*r*^2^=0.798, *p* ≤ 0.001).

**Conclusions:**

Our study illustrates a direct linear correlation between PAdP measured by CardioMEMS and simultaneous measurement of LV filling pressures derived by echocardiography.

## 1. Introduction

Congestive heart failure (CHF) impacts more than six million Americans. It is the admission diagnosis for more than one million hospitalizations annually and also accounts for the highest readmission rates [1, 2]. The socioeconomic burden associated with the management of heart failure is over $30 billion a year and is expected to exceed $70 billion annually by 2030 [3].

Increase in left atrial pressure (LAP) occurs earlier prior to any symptoms in the hemodynamic cascade of events that eventually leads to heart failure exacerbation [4]. Echocardiography is essential in evaluating the LAP; however, in certain conditions, the true estimate of LAP is limited by the presence of anatomical and physiological parameters [5]. In such circumstances, *E*/*e*' or Nagueh formula may not be the best method to calculate the LAP or pulmonary capillary wedge pressure (PCWP). In such instances, other diastology parameters such as isovolumetric relaxation time (IVRT) or IVRT/*T*_E-e'_ ratio are utilized to measure LAP. Furthermore, serial ambulatory evaluations by echocardiography are not only impractical but are also not cost-effective. Pressure-guided therapy is a novel strategy in the management of CHF which allows clinicians to proactively manage patients before they decompensate and require hospitalization. The CardioMEMS Heart Failure (HF) System is the only FDA approved implantable hemodynamic device, and provides real-time pulmonary artery (PA) hemodynamic data. Earlier investigations have reported a correlation between the standard PA-catheter measurements and echocardiography-derived estimates of pulmonary artery systolic pressure (PAsP) with CardioMEMS data [6]. We aimed to explore the agreement between simultaneous readings of pulmonary artery diastolic pressure (PAdP), a direct estimate of PWCP and LAP (assuming no diastolic pulmonary pressure gradient), obtained by the CardioMEMS HF system and noninvasive echocardiography-derived estimates of central hemodynamics in compensated HF patients.

## 2. Methods

### 2.1. Study Design

We prospectively enrolled seventeen patients with a history of CHF and preimplanted CardioMEMS HF device. Patients with presence of mitral prosthesis, severe mitral annular calcification, and permanent atrial fibrillation were excluded. Simultaneous CardioMEMS readings and a limited echocardiogram were performed at individual clinic visits to evaluate the agreement between PAdP and PAsP via CardioMEMS and echocardiography-derived estimates of LAP. The study was approved by the local institutional review board. All subjects provided an informed consent.

### 2.2. Data Collection

Subjects were brought in for a routine clinic visit. After initial clinical assessment for volume status, a limited echocardiogram was performed by one technician using a Philips Sonos 5500 with a 3.2 MHz transducer (Philips Medical Systems, Andover, MA), and three CardioMEMS readings were obtained and averaged. The limited echocardiogram included a parasternal long axis, right ventricular inflow and outflow, apical four chambers, and subcostal views for the inferior vena cava. All the four valves were pulsed to evaluate for stenotic and regurgitant lesions. Mitral inflow velocities were obtained from an apical four chamber view using the pulse wave Doppler. The sample volume was placed 1–3 mm between the mitral leaflet tips during diastole. The inflow velocities were measured at end-expiration with a sweep speed of 100 m/s. The tissue Doppler at the medial *e*' and lateral *e'* annulus was pulsed from an apical four chamber view. A sniff test was also performed at the time of evaluating the inferior vena cava (IVC) for estimation of right atrial pressure (RAP) [7]. PAsP was then calculated via the tricuspid regurgitation jet velocity (*V*_TR_) as follows: PAsP = 4(*V*_TR_)^2^ + RAP. Mitral inflow velocity (*E*) and septal and lateral annular (*e*') velocities were averaged to calculate the *E*/*e*' ratio. Estimated LAP was then calculated by taking the ratio of *E*/*e*' and applying the Nagueh formula (LAP = 1.24 × (*E*/*e*') + 1.9) [8]. Right heart catheterization tracings at the time of CardioMEMS implantation for all subjects were reviewed to assess for agreement between the PA pressure readings and presence of diastolic pulmonary gradient (DPG) defined as the difference between invasive PAdP and mean PCWP. PAdP correction was performed for DPG ≥ 7 mmHg at the time of CardioMEMS implant to adjust for presence of concomitant pulmonary vascular disease [9].

### 2.3. Statistical Analysis

All statistical analyses were performed using IBM SPSS, version 21.0. Summary statistics are presented as *N* (%) for categorical variables; continuous variables are presented as mean ± SD. Linear regression analysis was used for the comparison of PA pressures obtained with CardioMEMS and echocardiographic-derived measurements. Variability between the methods was expressed relative to the average PA pressures plus 2 SDs by Bland–Altman analysis. Significance was determined as a *p* value < 0.05.

## 3. Results

**Figure 1:**
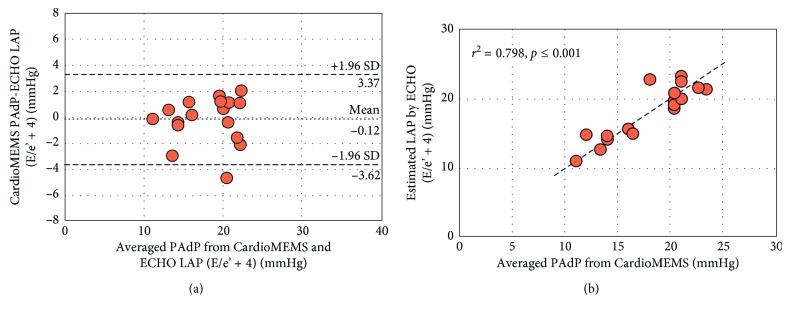
Bland–Altman analysis (a) and linear correlation (b) between CardioMEMS-derived pulmonary artery diastolic pressure (PAdP) and echocardiography-estimated left atrial pressure (LAP) by Nagueh formula (estimated LAP = 1.24 × (*E*/*e*') + 1.9).

**Figure 2:**
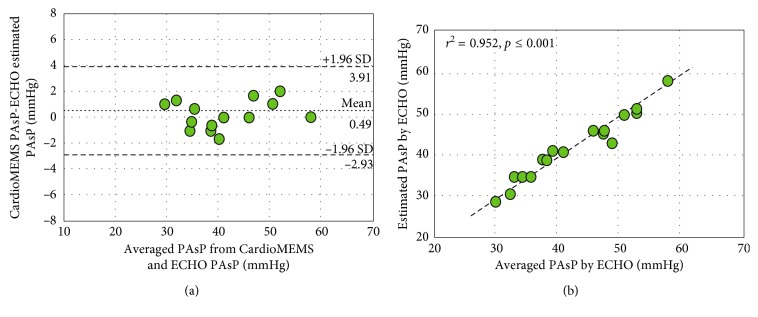
Bland–Altman analysis (a) and linear correlation (b) between CardioMEMS-derived pulmonary artery systolic pressure (PAsP) and echocardiography-estimated PAsP derived from tricuspid regurgitation jet velocity.

Baseline demographic, anthropomorphic, and clinical history are presented in Table 1. Baseline medical therapy for CHF at the time of assessment is presented in Table 1. Two subjects had baseline DPG ≥ 7 mmHg (8 and 11 mmHg) noted at the time of CardioMEMS implant requiring PAdP correction. All subjects were euvolemic by clinical exam and in normal sinus rhythm at the time of simultaneous echocardiographic assessment and CardioMEMS readings. Averaged invasive PA pressures from CardioMEMS implant date and simultaneous echocardiographic-derived and CardioMEMS hemodynamic data are presented in Table 2. Echocardiographic-derived LAP was higher compared to the PAdP reading from CardioMEMS (1.4 ± 2.1 mmHg, *p*=0.015) with no significant difference between estimated PAsP readings (0.49 ± 1.7 mmHg, *p*=0.264). Linear regression and agreement plots for PAdP and PAsP from CardioMEMS and echocardiogram as a function of average measurements are shown in Figures 1 and 2, respectively. A significant correlation was observed for the PAdP (*r*^2^=0.798, *p* ≤ 0.001) and PAsP (*r*^2^=0.952, *p* ≤ 0.001) measurements between both methods. Using Bland–Altman analytic methods, the bias for the echocardiographic estimates of the PAdP and PAsP was −0.12 mmHg and 0.49 mmHg with 95% limits of agreement ranging from +3.37 to −3.62 mmHg and +3.91 to −2.93 mmHg, respectively. Doppler echocardiography was inaccurate (defined as being greater than ±5 mmHg of the CardioMEMS measurement) in <1% (1/17) of cases for both PAdP and PAsP with PAdP being overestimated by 7 mmHg and PAsP underestimated by 6 mmHg by echocardiography in the aforementioned instances.

## 4. Discussion

Acute decompensated heart failure (ADHF) is associated with abnormal hemodynamics especially left ventricular filling pressure and cardiac output. While invasive hemodynamic monitoring using a pulmonary artery catheter in ADHF provides useful objective data, its clinical use has largely fallen out of favor in uncomplicated cases and is not recommended for routine hemodynamic monitoring in ADHF [10]. Echocardiography, on the other hand, is unarguably the most useful tool for initial assessment of patients with suspected diagnosis and follow-up of CHF allowing for risk-stratification of high-risk patients [11]. Progression of heart failure leads to chronic activation of neurohumoral regulation which promotes increase in atrial natriuretic peptide, brain natriuretic peptide, angiotensin II, and aldosterone all which contribute to left atrial remodeling [12]. These changes results in diastolic dysfunction which can be evaluated by Doppler echocardiography, and its presence has been associated with an increased all-cause mortality [13] and progression on serial assessments subsequent development of heart failure [14].

The use of *E*/*e*' is the most feasible and reproducible method for the estimation of filling pressures and predictive of normal or abnormal filling pressures when the ratio is <8 or >15, respectively [14, 15]. *E*/*e*' may be an unreliable way to predict LV filling pressures in certain clinical settings. For instance, those with long-standing HFrEF who have undergone left ventricular remolding may already have LV dilation and functional mitral regurgitation. The functional mitral regurgitation leads to an increase in transmitral inflow, the peak *E* velocity increases on the mitral inflow, and there is a reduction in the systolic to diastolic (S/D) ratio in the pulmonary venous flow [16]. In patients with decompensated heart failure or with cardiac resynchronization therapy, *E*/*e*' shows a poor correlation with intracardiac filling pressures especially with large LV volumes because of significant mitral regurgitation and wide QRS leading to abnormal septal motion [16]. Furthermore, in cases such as heart transplantation, mitral valve repair or replacement, severe mitral annulus calcification, or mitral stenosis, the annular tissue velocities are affected; therefore, *E*/*e*' is not the best indicator of LAP estimation [17]. Finally, poor acoustic windows may preclude accurate assessment of transmitral Doppler velocities.

Recognizing the potential limitations of transmitral Doppler velocities for assessment of LAP, the current American Society of Echocardiography/European Association of Cardiovascular Imaging guidelines for assessment of diastolic dysfunction recommend utilization of PAsP derived from *V*_TR_ as a surrogate marker for LAP in the absence of pulmonary vascular or parenchymal disease [15]. Again, the accuracy of the Doppler method for PAsP estimation is contingent upon obtaining the correct peak velocity from a coaxial *V*_TR_ signal from which the peak pressure can be estimated. A poor quality or noncoaxial signal can frequently underestimate the estimated PAsP. This occurs more than frequently in patients with severe lung disease where PAsP estimation should be avoided in the absence of a good *V*_TR_ envelope [16]. Chronic elevation in right sided filling pressures could lead to right ventricular (RV) compensation with decrease in tricuspid regurgitation and underestimation of PAsP [18]. Calculations using the *V*_TR_ Doppler signal assume that there is no pulmonary valve stenosis and may be inaccurate in the presence of RV systolic dysfunction. Furthermore, RAP is often overestimated if IVC measurement is used, leading to overestimation of PAsP [19].

Serial echocardiographic assessments provide little incremental prognostic information over clinical presentation or biomarkers in patients with ADHF [20]. In current practice, the role of echocardiography in the management of decompensated heart failure patients is limited to evaluation of LV function and regional wall motion abnormality. It is seldom used to guide therapy, and needless to say, monitoring of serial hemodynamics to prevent heart failure hospitalization is neither cost-effective nor realistic. In 2014, the U.S. FDA approved the CardioMEMS HF system which is a wireless, battery-free, PA pressure monitoring system. The device is implanted into a branch of the pulmonary artery during right heart catheterization and is powered by and interrogated via an external antenna. Pressures applied to the sensor causes deflections of the pressure-sensitive surface resulting in a characteristic shift in the resonant frequency. An external antenna achieves electromagnetic coupling. In the CHAMPION (CardioMEMS Heart Sensor Allows Monitoring of Pressure to Improve Outcomes in NYHA Class III Heart Failure Patients) trial, remote monitoring and titration of diuretic and vasodilator therapy based on patient-transmitted readings of PAdP from the CardioMEMS HF system showed a 37% reduction in heart failure-related hospitalizations (HR 0.63, 95% CI 0.52–0.77, *p* < 0.0001) [21].

There is only limited data on simultaneous assessment of PA pressures derived from the CardioMEMS device and echocardiographic estimates. Verdejo et al. performed serial assessments in 12 subjects to assess correlation between wireless PA pressure monitoring using CardioMEMS with Swan–Ganz catheter and echocardiographic PA pressure measurements [6]. CardioMEMS readings and echocardiography were performed at 2, 14, 30, 60, and 90 days while Swan–Ganz catheterization was performed at 0 and 60 days. The study showed an excellent correlation between all methods with high reproducibility and low interobserver variability between measurements. PAsP and PAdP measurements between the CardioMEMS device and Swan–Ganz catheter showed a significant correlation (*r*^2^=0.96 and 0.88 at baseline, *r*^2^=0.90 and 0.48 follow-up, *p* ≤ 0.01), with a mean difference of 6.2 ± 4.5 and −1.6 ± 6.8 mmHg, respectively. PAsP measurements between CardioMEMS and echocardiographic-derived estimate also had a significant correlation (*r*^2^=0.75, *p* ≤ 0.01) with a mean difference of −2.6 ± 11 mmHg. Echocardiography determination of PAdP in their study was only feasible in 2 patients due to technical reasons and hence was not reported.

Our study, for the first time, reports simultaneous hemodynamic readings from CardioMEMS and echocardiography-derived estimates of both PAsP and PAdP as a surrogate marker for LAP. Similar to the findings by Verdejo et al., there was a significant correlation between all CardioMEMS and Swan–Ganz catheter PA pressure readings at the time of implant amongst the enrolled patients on retrospective review of all tracings (data not presented). Of note, there were very few patients who had normal LV filling pressures because the cohort included patients with baseline NYHA class III heart failure who qualified for CardioMEMS implantation (Figure 1). Likewise, there was good correlation between CardioMEMS and echocardiographic-derived estimates of PAsP. While none of the subjects in our cohort had primary pulmonary hypertension, evidence of right ventricular dilatation/dysfunction or evidence of pulmonic stenosis, 9 (out of 17) subjects did have a history of emphysematous lung disease with 4 of them being on home oxygen therapy which, as stated above, could have potentially underestimated the echocardiographic-derived PAsP. This was indeed noticed in one of these subjects in our cohort (outlier, Figure 2(a)) with an inadequate *V*_TR_ signal and evidence of enlarged right atrium, signifying chronic elevation in right-sided filling pressures. In addition, there was presence of baseline DPG in 2 subjects signifying presence of mixed pulmonary hypertension. While Verdejo et al. could not reliably report echocardiography-derived PAdP, our study specifically aimed to look at its comparison with the CardioMEMS data. While there are currently no guidelines available for DPG correction for an accurate assessment of derived LAP, we used an acceptable DPG cutoff of 7 mmHg. All 17 subjects had reliable Doppler data for accurate assessment of estimated LAP with significant correlation seen between both methods.

## 5. Conclusion

Congestive heart failure is a syndrome that impacts millions of Americans on a daily basis and its management continues to evolve so that more patients are symptom-free, heart-failure hospitalization decreases, and socioeconomic burden associated with the management of heart failure is reduced. Chronic heart failure management is transitioning from symptom-guided therapy to pressure-guided therapy because the hemodynamic changes occur well in advance. The CardioMEMS HF system is the only FDA-approved device that has shown to reduce hospital readmission for heart failure. PAdP has now been established as the best guide for monitoring and titration of medications in ambulatory HF patients. Our study, for the first time, illustrates a direct linear correlation between PAdP measured by the CardioMEMS HF system and simultaneous measurement of echocardiography-derived estimated of LAP. While echocardiography remains as an invaluable tool in our clinical practice, one must be aware of the nuances associated with Doppler-derived estimates of filling pressures. CardioMEMS, in appropriately selected patients, is a valuable tool in a cardiologist's armamentarium to accurately monitor and optimize filling pressures in patients with CHF and reduce the risk of hospitalization.

## Figures and Tables

**Table 1:** Baseline characteristics.

	*N*=17
Age (mean ± SD)	74 ± 9
Gender (*n*, %)	
Male	10 (59%)
BMI (mean ± SD)	31.7 ± 6.3
Smoking (*n*, %)	
Never smoker	7 (41%)
Current smoker	1 (6%)
Former smoker	9 (53%)
CHF category	
HFrEF	15 (75%)
HFpEF	5 (25%)
Hypertension (*n*, %)	17 (100%)
Hyperlipidemia (*n*, %)	17 (100%)
Diabetes mellitus (*n*, %)	10 (59%)
Coronary artery disease (*n*, %)	14 (82%)
Peripheral arterial disease (*n*, %)	3 (18%)
Atrial fibrillation/other arrhythmias (*n*, %)	6 (35%)
Chronic lung disease (*n*, %)	9 (53%)
Chronic kidney disease (*n*, %)	4 (23%)

Baseline medical therapy for congestive heart failure	
Beta-blockers (*n*, %)	17 (100%)
ACE-I or ARB (*n*, %)	7 (41%)
ARB/neprilysin inhibitor (*n*, %)	8 (47%)
Aldosterone receptor blocker (*n*, %)	12 (71%)
Diuretics (*n*, %)	17 (100%)
Other vasodilators (*n*, %)	2 (12%)
Digoxin (*n*, %)	3 (18%)

**Table 2:** Invasive and noninvasive hemodynamic data from CardioMEMS implant and simultaneous echocardiographic assessment.

	*N*=17
Invasive hemodynamics at CardioMEMS implant	
PAsP, mmHg (mean ± SD)	49 ± 16
PAdP, mmHg (mean ± SD)	21 ± 7

Hemodynamics by CardioMEMS	
PAsP, mmHg (mean ± SD)	42 ± 8
PAdP, mmHg (mean ± SD)	18 ± 4

Estimated hemodynamics by echocardiogram	
PAsP, mmHg (mean ± SD)	42 ± 8
LAP, mmHg (mean ± SD)	19 ± 5

LAP, left atrial pressure; mPAP, mean pulmonary artery pressure; PAdP, pulmonary artery diastolic pressure; PAsP, pulmonary artery systolic pressure.

## Abbreviations

ADHF: Acute decompensated heart failure
CHF: Congestive heart failure
DPG: Diastolic pressure gradient
FDA: Food and Drug Administration
HF: Heart failure
IHD: Implantable hemodynamic device
IVC: Inferior vena cava
LAP: Left atrial pressure
LV: Left ventricle
NYHA: New York Heart Association
PA: Pulmonary artery
PAdP: Pulmonary artery diastolic pressure
PAsP: Pulmonary artery systolic pressure
PCWP: Pulmonary capillary wedge pressure
RAP: Right atrial pressure
RV: Right ventricle
VTR: Tricuspid regurgitation velocity.
